# ERGO: A pilot study of ketogenic diet in recurrent glioblastoma

**DOI:** 10.3892/ijo.2014.2382

**Published:** 2014-04-11

**Authors:** JOHANNES RIEGER, OLIVER BÄHR, GABRIELE D. MAURER, ELKE HATTINGEN, KEA FRANZ, DANIEL BRUCKER, STEFAN WALENTA, ULRIKE KÄMMERER, JOHANNES F. COY, MICHAEL WELLER, JOACHIM P. STEINBACH

**Affiliations:** 1 Dr. Senckenberg Institute of Neurooncology, University Hospital Frankfurt, D-60528 Frankfurt;; 2 Institute of Neuroradiology, University Hospital Frankfurt, D-60528 Frankfurt;; 3 Department for Neurosurgery, University Hospital Frankfurt, D-60528 Frankfurt;; 4 Institute of Physiology and Pathophysiology, Johannes Gutenberg-University, D-55099 Mainz;; 5 Department of Obstetrics and Gynecology, University Hospital of Würzburg, D-97080 Würzburg;; 6 Tavarlin AG, D-64293 Darmstadt, University Hospital Tübingen, D-72076 Tübingen, Germany; 7 Department of Neurology, University Hospital Tübingen, D-72076 Tübingen, Germany;; 8 Department of Neurology, University Hospital Zurich, 8091 Zurich, Switzerland

**Keywords:** feasibility, ketogenic diet, metabolism, glioma, glucose

## Abstract

Limiting dietary carbohydrates inhibits glioma growth in preclinical models. Therefore, the ERGO trial (NCT00575146) examined feasibility of a ketogenic diet in 20 patients with recurrent glioblastoma. Patients were put on a low-carbohydrate, ketogenic diet containing plant oils. Feasibility was the primary endpoint, secondary endpoints included the percentage of patients reaching urinary ketosis, progression-free survival (PFS) and overall survival. The effects of a ketogenic diet alone or in combination with bevacizumab was also explored in an orthotopic U87MG glioblastoma model in nude mice. Three patients (15%) discontinued the diet for poor tolerability. No serious adverse events attributed to the diet were observed. Urine ketosis was achieved at least once in 12 of 13 (92%) evaluable patients. One patient achieved a minor response and two patients had stable disease after 6 weeks. Median PFS of all patients was 5 (range, 3–13) weeks, median survival from enrollment was 32 weeks. The trial allowed to continue the diet beyond progression. Six of 7 (86%) patients treated with bevacizumab and diet experienced an objective response, and median PFS on bevacizumab was 20.1 (range, 12–124) weeks, for a PFS at 6 months of 43%. In the mouse glioma model, ketogenic diet alone had no effect on median survival, but increased that of bevacizumab-treated mice from 52 to 58 days (p<0.05). In conclusion, a ketogenic diet is feasible and safe but probably has no significant clinical activity when used as single agent in recurrent glioma. Further clinical trials are necessary to clarify whether calorie restriction or the combination with other therapeutic modalities, such as radiotherapy or anti-angiogenic treatments, could enhance the efficacy of the ketogenic diet.

## Introduction

The majority of solid tumors is characterized by increased glucose uptake and can therefore be detected by ^18^F-fluorodeoxyglucose positron emission tomography (FDG-PET). On a cellular basis, this is reflected by elevated glycolysis even in the presence of oxygen (aerobic glycolysis, the Warburg effect (1). There is also evidence of increased glycolysis in glioblastoma. First, malignant gliomas are characterized by activation of growth factor receptor/PI3 kinase/Akt signaling (2) leading to increased reliance on glycolysis (3) and by loss of p53 wild-type activity which can result in reduced expression of synthesis of cytochrome C oxidase 2 (SCO2), necessary for the proper assembly and function of the mitochondrial respiratory chain (4,5), and of tp53-induced glycolysis and apoptosis regular (TIGAR), which suppresses glycolysis (6,7). Second, hypoxia typically present in malignant glioma is expected to stimulate accumulation of HIF-1α and subsequent expression of genes involved in glucose metabolism and in the suppression of oxidative phosphorylation (8, 9). Indeed, there is evidence of HIF-1α and glucose transporter 3 (GLUT3) expression (10) and of increased lactate accumulation in malignant gliomas (11). Further, FDG-PET and ^18^F-fluoromisonidazole (FMISO)-PET showed increased glucose uptake and hypoxia in malignant gliomas (12,13). Recently, innovative metabolic flux analyses confirmed increased glucose metabolism in glioblastoma tissue (14) and mouse xenograft tumors (15). Interestingly, glucose is metabolized not only to lactate, but also via the tricarboxylic acid (TCA) cycle (14–16) possibly providing proliferating cells with carbon precursors for anabolic metabolism. Additionally, glucose is metabolized by the pentose phosphate pathway thereby providing riboses for nucleic acid synthesis and producing NADPH which is involved in antioxidative defense mechanisms (17).

Apart from the tumor-intrinsic consequences of increased glycolysis, glucose metabolism of the whole organism seems to affect tumor growth. For example, elevated levels of insulin are associated with worse prognosis in breast cancer patients (18), and increased insulin-like growth factor-1 (IGF-1) levels are associated with an elevated risk of prostate (19,20) and breast cancer (21). These observations may relate to the fact that insulin and IGF-1 not only modulate glucose metabolism of healthy tissues but also act as growth factors for tumor cells. The influence of insulin on tumor formation and growth is supported by epidemiologic analyses where tumor rates were higher in diabetic patients treated with insulin-releasing drugs such as sulfonylureas or with insulin, but not in patients treated with metformin, which does not increase insulin levels (22). Supporting the assumption that glucose is also important for glioma growth and therapy resistance, higher blood glucose levels are associated with worse prognosis in patients with glioblastoma (23).

Therefore, reducing glucose availability by dietary restriction of glucose and carbohydrates might affect tumor growth. Such a restriction can be achieved by a ketogenic diet, characterized by low carbohydrate intake and high fat and balanced protein content. In this situation, ketone bodies such as acetoacetate and 3-hydroxybutyrate (3-OHB) are produced which serve as alternative energy substrates for brain cells. In different murine xenograft models, ketogenic diets inhibit tumor growth (24–26). Clinically, the ketogenic diet is an effective treatment for children and probably also for adults with refractory epilepsy (27). These diets reduce body weight in obese patients, modulate blood lipid profiles (28), and might decrease blood levels of glucose, insulin and IGF-1 (29,30). Only few studies on ketogenic diets in tumor patients exist. A description of two pediatric patients with anaplastic glioma indicated that a ketogenic diet might reduce tumor glucose uptake and inhibit tumor growth (31). Two studies on patients with different solid tumors showed no severe side-effects of ketogenic diets (32,33). No prospective registered clinical study on feasibility, safety and efficacy of a ketogenic diet in a specific tumor type and in glioma in particular has been reported up to now. Considering the plausible rationale for the ketogenic diet and the lack of established treatment options for recurrent glioblastoma, the ERGO study was set up to investigate safety and tolerability of an unrestricted ketogenic diet in patients with recurrent glioblastoma.

## Materials and methods

### Ethics statement

The study was approved by the local Institutional Review Boards of the Frankfurt (no. 113/08) and Tübingen (no. 338/2007BO1) University hospitals. The animal experiment protocol was approved by the Regierungspräsidium Darmstadt (no. F 145/01).

### Study design

This trial included patients with recurrent glioblastoma. Important inclusion criteria were age ≥18 years, detection of relapse ≥6 months after initial tumor surgery and ≥3 months after completion of radiotherapy, relapse during or after temozolomide chemotherapy, no other reasonable chemo-therapeutic option or chemotherapy refused by the patient and Karnofsky performancy score (KPS) of ≥60%. There were no restrictions concerning values of whole blood cell counts at inclusion. Important exclusion criteria were diabetes mellitus requiring insulin treatment or decompensated cardial insufficiency. The ERGO trial was an open-label, prospective, single-arm pilot study performed at the Frankfurt and Tübingen University Hospitals. The study was registered at www.clinicaltrials.gov (NCT00575146). Patients were recruited between December 2007 and March 2010. Follow-up was until November 2011. All patients gave their written consent before study inclusion. At baseline and follow-up visits in 6–8-week intervals or if signs of clinical progression occured, medical history, history of seizures, KPS, mini-mental status, quality of life questionnaire, neurological examination, vital signs, laboratory parameters, adverse events and medication were assessed.

The primary endpoint was feasibility of the ketogenic diet defined as percentage of patients who discontinued diet due to intolerability, secondary objectives were safety of the diet, the percentage of patients reaching ketosis, quality of life, progression-free survival (PFS) and overall survival.

### Treatment

**Figure 1.**
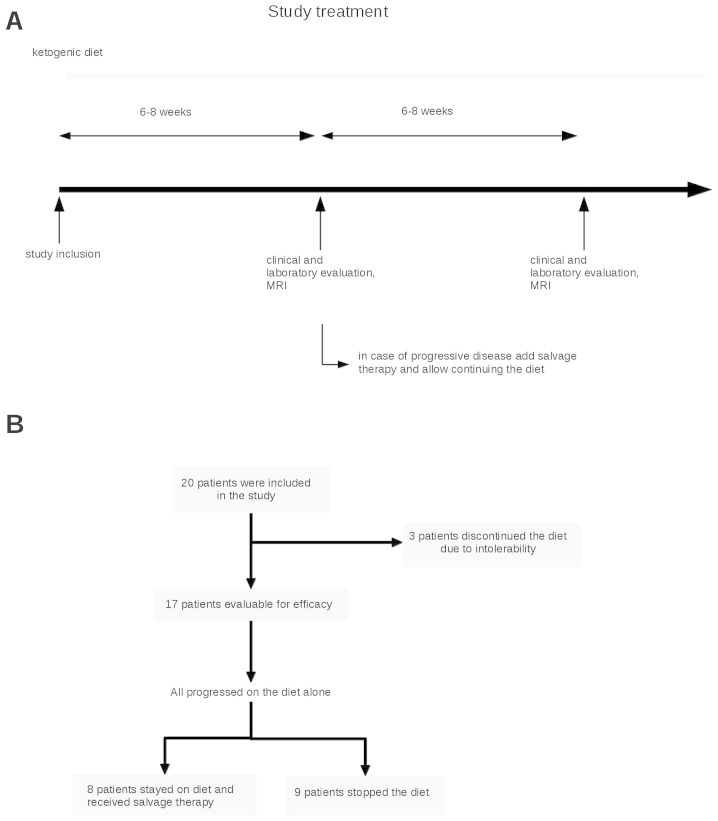
Study design and course of treatment. (A) After study inclusion, patients were treated with the ketogenic diet. At 6 weeks on the diet or in the presence of clinical signs of deterioration, MRI was performed. In case of at least stable disease, diet was continued. In case of a progression, the patients were offered salvage therapy while continuing the diet. (B) Flowchart diagram showing the course of dietary treatment in the 20 included patients.

The patients were put on a ketogenic diet which restricted carbohydrate intake to 60 g/day. In addition, highly fermented yoghurt drinks (500 ml per day) and two different plant oils (basic oil and addition oil) were provided to the patients and could be consumed on an individual basis. The drinks contained 2.42 kJ/g (0.01 g carbohydrates/g, 0.04 g fat/g and 0.02 g protein/g), the energy content of the oils was 37.3 kJ/g (0 g carbohydrates/g, 0.99 g fat/g, 0.0 g proteins/g). No calorie restriction was applied, and patients were instructed to always eat to satiety. Support for the implementation of the diet, drinks and oils were provided by Tavarlin (Darmstadt, Germany). Before starting the diet, the patients were introduced into the principles of the ketogenic diet, and a set of brochures with sample cooking recipes and food facts as well as the basic rules to follow the diet were provided. The patients thereafter indivually prepared their meals at home, no standarized eating plans were provided. The patients self-monitored urine ketones 2–3 times per week using urine test sticks (Ketostix, Bayer, Germany) and filled-out a nutritional plan. Further, the patients were asked to complete a questionnaire covering the following aspects every week: diarrhea, constipation, hunger and demand for glucose. These items were rated from 0 (none) to 3 (strong). After 6–8 weeks or signs of clinical progression, disease status was assessed by magnetic resonance imaging using Macdonald criteria (34). In case of stable disease or response, patients were to continue the diet (Fig. 1A). In case of progression, the protocol allowed to continue the diet while salvage therapy was initiated. In case of further progression on a combination, the diet was stopped. Further treatment was at discretion of the caring physician.

### Animal experiments

The high response rate in patients exposed to bevacizumab upon progression while maintaining the diet led us to perform an exploratory trial on the combination of the ketogenic diet and bevacizumab in the U87MG model: 44 female 7-week-old athymic mice (HSD:athymic nude-Foxn1nu, Horst, The Netherlands) were maintained in groups of 3–4 animals per cage in a pathogen-free environment and given *ad libitum* access to food and water. All animal work was performed in accordance with the National Institutes of Health guidelines Guide for the Care and Use of Laboratory Animals and institutional standards. The protocol was approved by the Regierungspräsidium Darmstadt (no. F 145/01). On day 0 of the experiment, 10^5^ human U87MG glioma cells were stereo-tactically implanted into the right striatum. On day 7, animals were randomly assigned to either an unrestricted standard diet rich in carbohydrates or an unrestricted ketogenic diet. The standard diet was provided by the animal feed manufacturer ssniff Spezialdiaeten GmbH (Soest, Germany), the ketogenic diet was prepared on the basis of KetoCal^®^ Advance (Nutricia GmbH, Erlangen, Germany). Diet characteristics are summarized in Table I. Starting on day 12, bevacizumab (10 μg/g body weight, Roche, Basel, Switzerland) or phosphate-buffered saline (PBS, control) were administered intraperitoneally twice weekly. On day 28, 12 animals, 3 per group, underwent MRI imaging and were subsequently sacrificed for metabolic bioluminescence imaging. The remaining 32 animals were sacrificed at the onset of neurological symptoms or weight loss of >20% of the body weight.

### Magnetic resonance imaging (MRI) of animals

Imaging was performed in prone position on day 28 after tumor cell implantation at a 3-Tesla MRI scanner (Trio^®^, Siemens, Erlangen, Germany) using a circular polarized wrist coil and 0.5 mmol/ml gadolinium-diethylenetriaminepentaacetic acid (Magnevist^®^, Bayer Schering Pharma, Berlin, Germany). Coronar T2-weighted and T1-weighted sequences were acquired with a slice thickness of 2 mm without gap and an inplane resolution of 0.2×0.2 mm. Imaging was performed after intraperitoneal injection of 0.3 ml of 0.5 mmol/ml gadolinium-diethylenetriaminepentaacetic acide (Magnevist^®^, Bayer Schering Pharma, Berlin, Germany). The largest perpendicular diameters of the contrast-enhancing tumor in the three dimensions were determined, and the tumor size was estimated using the ellipsoid volume formula π/6 × length × width × depth.

### Determination of blood 3-OHB

Blood 3-OHB levels of randomly chosen animals (5–7 animals per group) were measured on day 24 in 2 μl of peripheral blood from the tail vein using a Precision Xtra^®^ monitoring system (Abbott Laboratories, Abbott Park, IL, USA).

### Metabolic mapping using bioluminescence imaging

Bioluminescence imaging indicating local concentrations of the metabolites ATP, lactate and glucose in cryosections from rapidly frozen brains (3 animals per group) was performed as previously described (35,36).

### Statistical analysis

Data analysis was carried out with SPSS version 17.0 (IBM SPSS, Chicago, IL, USA). Significance was tested using the Mann-Whitney U test. Survival was estimated by Kaplan-Meier analysis, and differences were tested by Mantel-Cox log-rank statistics.

## Results

### Baseline characteristics

Twenty patients were enrolled between December 2007 and March 2010. Baseline characteristics and pretreatment modalities of the patients are summarized in Table II. All patients had a histological diagnosis of glioblastoma. Primary therapy included radiotherapy with 60 Gy in all patients. In 16 patients (80%), radiotherapy was combined with concomitant temozolomide. Eighteen patients (90%) were pretreated with dose-dense temozolomide (‘one week on/one week off’). One patient was treated with bevacizumab and lomustine prior to study inclusion. The median number of relapses, including the relapse leading to study inclusion, was 2 (range 1–4). The median time from the initial diagnosis of glioblastoma to the start of the study treatment was 12.5 months (range, 6–42 months).

### Feasibility

**Figure 2.**
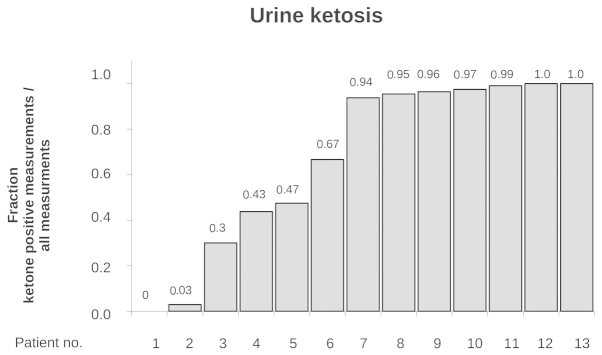
Urine ketosis. The fraction of ketone body-positive urine analyses (ketone bodies >0.5 mmol/l) is reported in the 13 patients with these tests available. Each bar represents the value of one patient, sorted from lowest to the highest ratios (left to right). The individual values are also presented above each bar.

Three patients discontinued the diet in the absence of progression after 2–3 weeks mainly because they felt that carbohydrate restriction negatively affected their quality of life (Fig. 1B). Of the remaining 17 patients who stayed on diet at least until tumor progression, clinical and laboratory parameters before and at follow-up at a median of 36 days on study treatment are shown in Table III. There was a small, statistically significant weight loss of ∼2.2% during the diet. A regular urine ketone analysis (at least twice a week) during the dietary treatment was available in 13 patients, and ketosis was detectable at least once in 12 of these patients (92%). In these, an average of 73% of the measurements documented ketonuria indicating rather stable ketosis in the majority of patients (Fig. 2).

### Safety and tolerability

**Figure 3.**
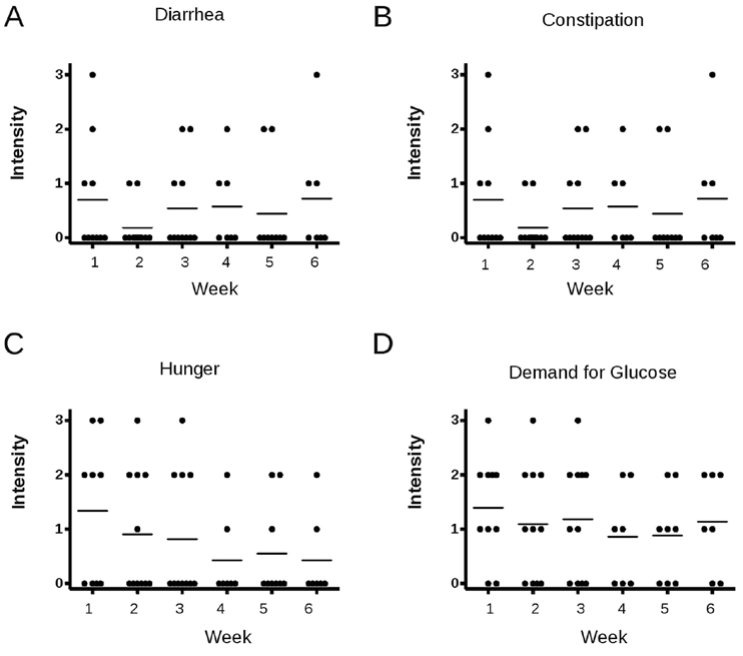
Patients’ self reported rating of diet tolerability. The patients were asked to fill out a self-reported questionnaire for possible diet-related side-effects once every week on diet. These sheets were completed at least once in 12 patients. Shown are the ratings of each patient in the categories diarrhea (A), constipation (B), hunger (C) and demand for glucose (D) in every week (dot) and the mean value of these ratings in the corresponding week (line). The scale was defined as follows: 0, not present; 1, weak; 2, moderate; 3, strong. Questionnaires were available in 7–11 patients at the shown time-points.

At least one self-reporting sheet on possible diet-related side-effects was available in 12 patients. Patients stated that they followed the diet on an average of 6.8 days per week. No serious adverse events possibly attributable to the diet, i.e., hypogylcemia, occured. The majority of the patients did not complain diarrhea or constipation (Fig. 3A and B). Hunger was present in the first week on diet at a mean intensity of slightly >1 (which means weakly feeling hungry) and decreased on the following weeks. A similar pattern was observed for appetence for sugar (Fig. 3C and D).

At baseline, grade 3 leukocytopenia was present in 2 patients and at follow-up in 1 of these patients. No other grade 3 toxicity was observed during the study period. No significant changes in laboratory parameters, including blood glucose and HbA1c values, occurred during the diet in any of the analyzed parameters (Table III).

### Efficacy

**Figure 4.**
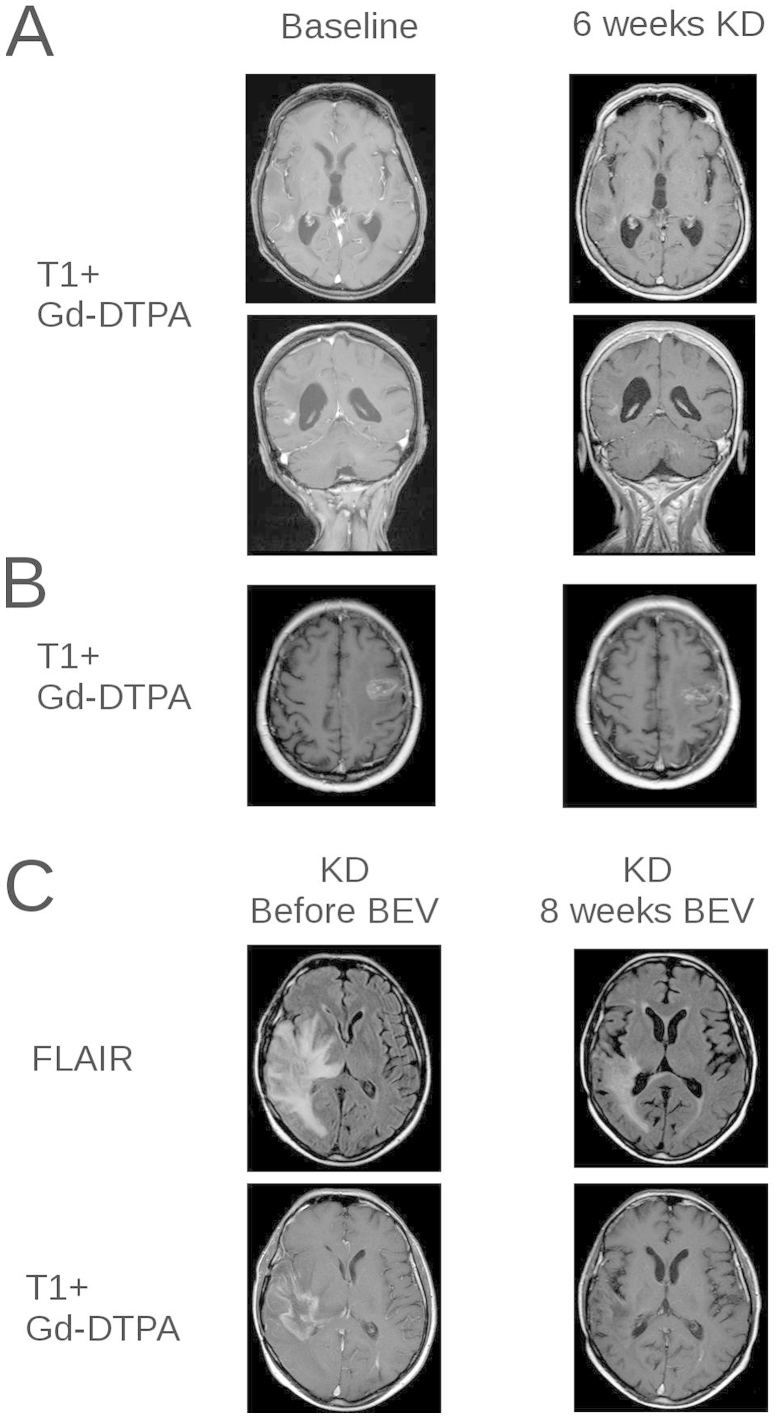
MRI finding in 3 patients. (A) T1-weighted images after intravenous application of gadolinium-DTPA (Gd-DTPA) before (left panel) and after 6 weeks on the ketogenic diet (KD) (right panel) in axial (upper) and coronar (lower) planes of the patient with a minor response. (B) T1-weighted images after intravenous application of Gd-DTPA before (left panel) and after 6 weeks on the ketogenic diet (right panel) in axial directions of a patient with stable disease. (C) Fluid-atenuated inversion recovery (FLAIR) images and T1-weighted images after application of Gd-DTPA before (left panel) and 6 weeks after start of bevacizumab (BEV) in a patient with partial response to bevacizumab who continued the diet.

**Figure 5.**
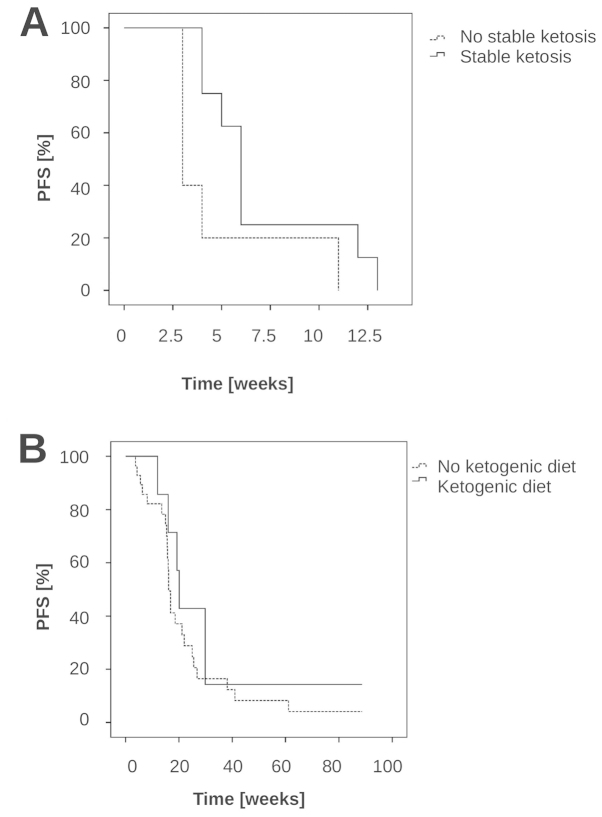
Analysis of PFS. (A) PFS of patients on diet who achieved stable ketosis as defined as urine ketosis in >50% of measurements (n=8) compared to those patients who did not achieve stabile ketosis (n=5) was analysed by Kaplan-Meier analysis (p=0.069, log-rank-test). (B) PFS of patients who received bevacizumab while on ketogenic diet (n=7) vs. a cohort of patients who were treated in the same period of time with bevacizumab but without ketogenic diet (n=28) (p=0.38, log-rank test).

Median time to progression on the diet was 5 weeks (range, 3–13 weeks). In 3 patients, stable disease was observed at first follow-up at 6 weeks, and stabilization lasted for 11, 12 and 13 weeks in these patients; one patient achieved a minor response (Fig. 4A and B). Median overall survival after start of the diet was 32 weeks (range, 6–86+ weeks). We further analysed whether stable ketosis might be associated with PFS. There was a trend for longer PFS in the group who had stable ketosis compared to the other patients (Fig. 5A) (median PFS stable ketosis (n=8) 6 weeks vs. no stable ketosis (n=5): 3 weeks, p=0.069, log-rank-test). To obtain preliminary insights into the tolerability and efficacy of the diet in combination with other therapies, the study protocol allowed the addition of a salvage therapy at first progression while continuing the diet. Progression was documented in all of 17 patients on diet. Thirteen of these received no salvage treatment. Eight patients continued diet with the salvage treatment consisting of ACNU/teniposide in 1 patient and bevacizumab alone (n=4) or in combination with irinotecan (n=3). Among these 7 patients there were 1 complete response and 5 partial responses (Fig. 4C), for an overall response rate of 85%. Median PFS from bevacizumab was 20.1 weeks (range, 12–124 weeks). PFS at 6 months (PFS-6) was 43%. We compared these results with a cohort of 28 patients who were treated with bevacizumab in the same period in our institution, but who were not on a ketogenic diet. In these, median PFS was 16.1 weeks (range, 4–90+ weeks; 95% CI, 15–17 weeks), p=0.38 (log-rank-test compared to the ketogenic diet + bevacizumab-treated patients) (Fig. 5B), and the response rate was 65% (17/26 evaluable patients), (comparison of the response rates bevacizumab and diet vs. bevacizumab: p=0.4, Fisher’s exact test).

### Combination of a ketogenic diet and bevacizumab in an orthotopic glioma model

**Figure 6.**
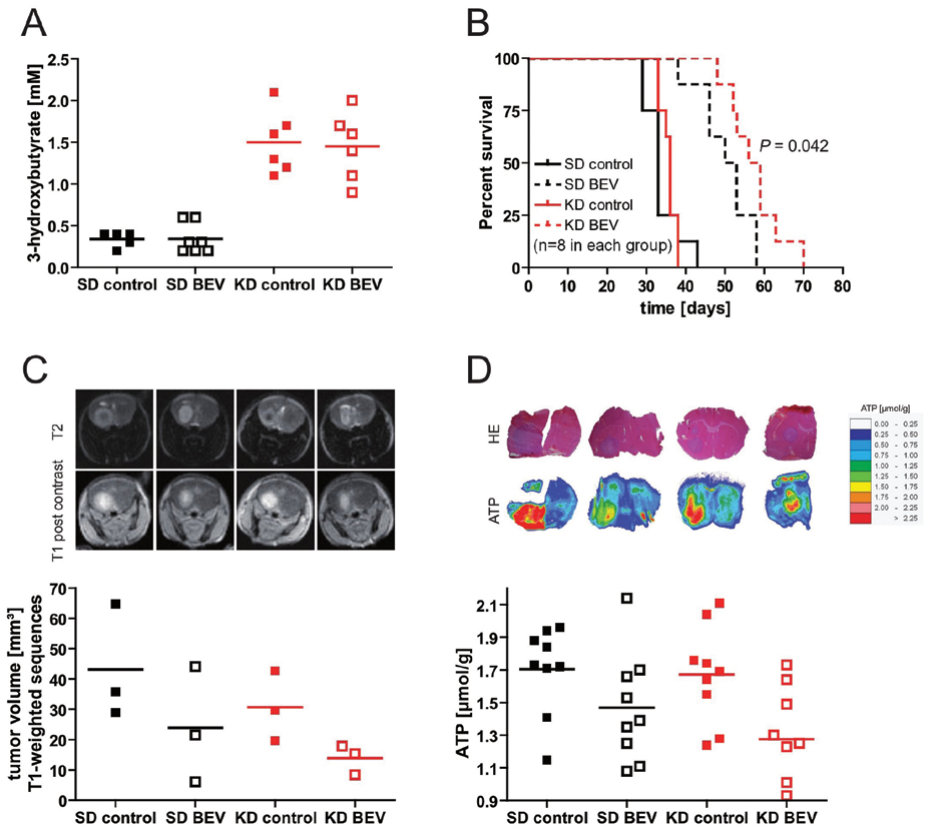
Mouse xenograft experiments. (A) 3-OHB levels in the blood were determined 24 days after U87MG tumor cell inoculation in mice on standard diet (SD) or ketogenic diet (KD) treated with PBS (control) or bevacizumab (BEV). (B) Kaplan-Meier analysis of the treatment groups. (C) At day 28 after tumor cell inoculation, 3 mice per group were analysed by MRI. Upper panel, representative T2-weighted images and T1-weighted images after infusion of Gd-DTPA are shown. Lower panel, tumor volumes were measured as described, and individual (dot) and mean values (bar) are indicated. (D) Upper panel, representative hematoxylin-eosin stainings (H&E) and color-coded distribution of ATP concentrations in representative slices. Lower panel, ATP content in the tumor areas was quantified, and individual (dot) and mean values (bar) are shown.

The high response rate to bevacizumab in patients progressing on the diet led us to explore whether a low carbohydrate, ketogenic diet would modulate the efficacy of bevacizumab in the U87MG orthotopic glioma model. The ketogenic diet led to a significant elevation of 3-OHB levels (Fig. 6A) indicating pronounced ketosis in the animals fed the ketogenic diet. Basal glucose levels were not different between the two diet groups (not shown). Importantly, whereas the ketogenic diet alone had no significant effect on survival, the combination of ketogenic diet and bevacizumab prolonged survival compared to bevacizumab alone (median survival 52 vs. 58 days, p<0.05, log-rank test, Fig. 6B). Tumor volumes analysed by MRI similarly tended to be smaller with the combination (standard diet + bevacizumab, 23.9 mm^3^ vs. ketogenic diet + bevacizumab, 13.8 mm^3^; p>0.05) (Fig. 6C). To investigate whether the ketogenic diet modulated metabolic parameters, the contents of glucose, lactate and ATP in three tumor tissue slices in three mice per group was analysed. While there was no significant difference in glucose and lactate between groups, treatment with bevacizumab signficantly reduced ATP levels within the tumor tissue in both diet groups (standard diet vs. standard diet + bevacizumab: p=0.047; ketogenic diet vs. ketogenic + bevacizumab p=0.017), and there was again a trend for a stronger effect of bevacizumab in the ketogenic diet-treated animals (Fig. 6D).

## Discussion

In the present study, the unrestricted ketogenic diet was safe and relatively well tolerated (Table III, Figs. 2 and 3). Three patients discontinued the diet in the absence of tumor progression for poor tolerability. In the remaining 17 patients, ketosis was achieved in 12 of 13 evaluable patients. In two patients, despite apparently strong adherence to diet as monitored by the nutritional questionnaires, no or nearly no ketosis (ketosis in 0 and 3% of measurements, Fig. 2) was achieved, indicating that there might be genetic or other unknown factors affecting the shift to a ketotic state by the applied unrestricted ketogenic diet. No severe toxicity was observed, as indicated by the absence of serious diet-related adverse events and unchanged routine laboratory parameters. Furthermore, weight loss, although being statistically significant, was only weak.

The limited number of patients, absence of randomization and lack of a control group in the study do not allow an unequivocal estimation of efficacy of the ketogenic diet. However, it appears that single agent activity, if any, in these heavily pretreated patients was moderate at best. Although three patients achieved a stable disease at 6 weeks, median PFS was only 5 weeks, and PFS at 6 months was 0%.

One important reason for the low clinical activity might be the failure to significantly lower glucose levels by the ketogenic diet (Table III). Causes for it may involve the frequent use of steroids in these patients (Table III) and the fact that no calorie restriction was applied. Another explanation might be the assumption that tumor cells could circumvent reduced glucose availability by the use of ketone bodies. However, we previously showed that glioma cell lines, in contrast to rat hippocampal neurons, are not capable of metabolizing ketone bodies (36). Accordingly, a recent study showed that the expression levels of the ketone body-metabolizing enzymes succinyl-CoA 3-oxoacid CoA transferase (OXCT1) and 3-hydroxybutyrate dehydrogenase 1 (BDH1) are reduced in glioma tissue (37).

Because ketone body metabolism requires oxygen for energy production via oxidative phosphorylation, and because hypoxic tumor cells are more susceptible to glucose restriction (38), the ketogenic diet could provide hypoxic tumor areas with a specific disadvantage concerning energy metabolism. Prolonged anti-angiogenic treatment probably increases hypoxia as reflected by upregulated expression of HIF-1α and carbonic anhydrase 9 (39–41) and by a decrease of T2’ values in MRI indicative of a higher proportion of deoxyhemoglobin (42–44). Combining low-carbohydrate diets with these therapies could therefore act synergistically. Although patient numbers are small, we noted a high response rate to bevacizumab in patients on the diet that may or may not have been achieved with bevacizumab alone. The results of the mouse model suggest increased efficacy of the combined treatment (Fig. 5). A significant decrease of ATP levels accompanied by unaltered glucose concentrations in the tumor tissue of bevacizumab-treated mice could indicate that glucose levels in the tumor are insufficient to sustain ATP production. One reason might be a lack of oxygen leading to less efficient ATP generation by suppressed oxidative phosporylation and therefore increased glucose needs (5,7,38). Microdialysis analyses have shown a correlation between systemic glucose concentrations and glucose levels within the glioma tissue (11). Although in our and other mouse models (45) basal blood glucose levels remained unchanged between the different diet groups, postprandial glucose peaks could be reduced by carbohydrate-restricted meals (46). The prevention of transient glucose excess may therefore be a mechanism contributing to the enhancement of bevacizumab’s efficacy by the ketogenic diet in in the mouse xenograft experiments and the trend in the combination-treated patients. As peak glucose concentrations were not determined in the patients of the ERGO study, this explanation remains speculative, however. Alternatively, since ketogenic diet may have pleiotropic effects on tumor cells or surrounding glia cells, it may cause a more bevacizumab-sensitive phenotype via a yet-to-be-defined metabolic switch or alterations in the tumor microenvironment.

Various attempts have been made to enhance the efficacy of a ketogenic diet. Although activity of an unrestricted ketogenic diet alone has been described in the GL-261 glioma model (26), calorie restriction was required for efficacy in the CT-2A glioma model (24,47). Calorie restriction is known to inhibit tumor growth in a variety of other xenograft tumor models (48–50). In the ERGO study, no calorie restriction was applied considering that it might be unethical to continuously reduce calorie intake for several weeks in tumor patients in a palliative situation. However, given the wealth of preclinical experiences, the clinical efficacy of the ketogenic diet might be increased even by transient calorie restriction. In addition, the combination of calorie restriction or fasting with other therapeutic modalities such as chemotherapy or radiotherapy is effective in mouse xenograft models (51,52). As a first, preliminary indication of feasibility and efficacy, an impressive response has been reported in a glioma patient who received radiotherapy and chemotherapy together with a calorie-restricted ketogenic diet (53). Further randomized clinical trials are warranted to clarify whether calorie-resticted ketogenic diets might be clinically efficient antitumor strategies.

In conclusion, we report that the ketogenic diet can be safely applied to glioblastoma patients. Pilot animal data indicate increased acitivity of bevacizumab when combined with the ketogenic diet. Additional research on the mechanisms of the diet combined with antiangiogenic or vascular targeted treatments or conventional therapies are necessary to clarify a possible role of the ketogenic diet for glioblastoma therapy.

## Figures and Tables

**Table I.** Composition of the standard and ketogenic diets for the animals.

Component	Standard diet	Ketogenic diet
Fat	6.1	56.1
Carbohydrate	55.6	2.9
Protein	21.8	15.0
Fiber	3.8	1.7
Energy (kJ/g)	15.8	23.8
Ketogenic ratio	0.08:1	3.14:1

Components of the diets used are listed in grams (g) per 100 g of food. The fat in both diets derived from soybean oil. The ketogenic diet was based on KetoCal Advance, a nutritionally complete formula used for children with intractable epilepsy, and supplemented with flaxseed and egg white. The ketogenic ratio was calculated according to the following formula: fats/(protein + carbohydrates).

**Table II.** Baseline characteristics.

Age (years)	57 (30–72)
Gender	13 female, 7 male
Number of relapses	2 (1–4)
Karnofsky performance score	85 (70–100)
Previous treatments	
Radiotherapy	20 (100%)
Concomitant temozolomide	16 (80%)
Temozolomide 5/28	14 (70%)
Temozolomide 7/14	18 (90%)
Nitrosourea-based chemotherapy	5 (25%)
Carmustine wafer	1 (5%)
Bevacizumab + lomustine	1 (5%)

Presented are patient characteristics at study entry. For age, number of relapses and KPS, median and range are presented. For previous therapies, the numbers and percentages of patients that received the denoted treatment before study entry are shown.

**Table III.** Clinical and laboratory parameters during the study.

	Before diet	During diet
Clinical parameters		
Weight (kg) (mean ± SD)	78.3±16.1	76.5±14.6
Mean weight difference (kg)		−1.86^a^
(%)		−2.2%
Steroids		
No	9	6
Yes	8	11
Median dexamethasone dose in mg (range)	4 (2–20)	8 (2–24)
Blood		
Glucose (mg/dl) (mean ± SD)	99±21.8	92±9.1
No steroids, n=5	98±29.1	92±5.8
Steroids, n=4	97±19.1	90±9.3
HbA1c (%) (mean ± SD)	5.42 ±0.48	5.60±0.35
Triglycerides (mg/dl) (mean ± SD)	156±69	131±56
Cholesterol (mg/dl) (mean ± SD)	228±41	222±51
HDL (mg/dl)	60±18	61±17
LDL (mg/dl)	136±36	134±39
HDL/HDL quotient	0.50±0.28	0.49±0.17

Presented are clinical and laboratory parameters at baseline and at first follow-up visit. Paired weight values were available in 14 patients (^a^ p<0.05, paired t-test), paired blood parameters were obtained in 11 patients. Glucose values are separately shown for the 5 patients not on steroids at baseline and at first follow-up, and also for the 4 patients on steroids at both visits.

## Abbreviations:

3-OHB: 3-hydroxybutyrate;
BEV: bevacizumab;
FDG-PET: ^18^F-fluorodeoxy-glucose positron emission tomography;
FMISO: ^18^F-fluoromisonidazole;
HIF-1α: hypoxia-inducible factor-1α;
IGF-1: insulin-like growth factor-1;
KPS: Karnofsky performance score;
MRI: magnetic resonance imaging;
PFS: progression-free survival;
PI3 kinase: phosphatidyl-inositol-3 phosphate kinase;
ROS: reactive oxygen species;
SCO2: synthesis of cytochrome C oxidase;
TCA cycle: tricarboxylic acid cycle;
TIGAR: tp53-induced glycolysis and apoptosis regulator
